# Supplementary material for: Overtone photothermal microscopy for high-resolution and high-sensitivity vibrational imaging
Source: Nat Commun. 2024 Jun 25;15:5374. doi: 10.1038/s41467-024-49691-2 (PMC11199576; doi:10.1038/s41467-024-49691-2)
Supplement: Supplementary file 1 — Supplementary Information [file 41467_2024_49691_MOESM1_ESM.pdf]

Supplementary Information

Overtone photothermal microscopy for high-resolution and high-sensitivity vibrational imaging

Le Wang,[1,†] Haonan Lin,[1,†] Yifan Zhu,[2] Xiaowei Ge,[1] Mingsheng Li,[1] Jianing Liu,[1] Fukai Chen,[3] Meng Zhang,[1] and Ji-Xin Cheng[1,2,3]*

[1]Department of Electrical and Computer Engineering, Boston University, Boston, MA, 02215, USA
[2]Department of Chemistry, Boston University, Boston, MA, 02215, USA
[3]Department of Biology, Boston University, Boston, MA, 02215, USA

†These authors contributed equally to this work.

*Corresponding author: jxcheng@bu.edu

Note S1. Procedures and parameters of COMSOL simulation of the thermodynamic traces of 200-nm PMMA beads

We utilized the software COMSOL Multiphysics 6.0 to calculate the transient temperature change, i.e., thermodynamic traces, of 200-nm PMMA beads upon SWIR excitation. Our computational approach followed the methodology previously established by Li *et al.* for mid-infrared photothermal microscopy.[1] The investigation focused on analyzing the thermodynamic traces of PMMA beads immersed in two different media, glycerol-d8 and D_2O.

Taking glycerol-d8 as an example for the illustration of simulation parameters, we first calculated the absorption cross-section σ_{abs} of 200-nm PMMA beads at 1170 nm with a standard Mie scattering model (https://omlc.org/calc/mie_calc.html). The known refractive index of PMMA at 1170 nm is 1.48 + 0.0005i,[2] and the real part of the refractive index of the glycerol-d8 is 1.46. With these optical parameters and the bead size, σ_{abs} was numerically determined to be $\sigma_{abs} = 2.26 \times 10^{-5}$ µm^2.

The SWIR heating beam has a repetition rate of 80 MHz and was treated as a pseudo-continuous wave. Each pulse train after modulation was modeled as a rectangular function. To align with experimental conditions, the modulation frequency of the SWIR beam was set to 800 kHz, with a duty cycle of 50%, equivalent to a pulse width of 625 ns. The input average power of the heating beam was 40 mW, corresponding to a SWIR energy of 50 nJ and a peak power of 80 mW. The total absorbed SWIR power P_{abs} is proportional to the SWIR intensity and the absorption cross-section. From the area of the focal spot and the SWIR peak power, we evaluated the intensity value at the center as 4.9×10^{10} W/m^2.

To simulate the heat dissipation of the beads, we utilized COMSOL's heat-transfer-in-solid model. Our model consisted of a 200-nm PMMA bead deposited on the surface of a cover glass, with glycerol-d8 or D_2O serving as the surrounding medium for a comparison of thermal behaviors under different conduction conditions. The diameter of the cover glass and the dispersion media was set to be 60 times larger than that of the PMMA beads to ensure that heat dissipation was not affected by the boundaries. We assumed a continuous temperature profile at the PMMA-glass interface and the PMMA-medium interface. The initial temperature was set as room temperature 293 K, and the temperature increase ΔT was subsequently calculated to generate the thermodynamic traces. The heat source was set to the whole PMMA bead, and for simulation simplicity, we assumed a uniform distribution of heating power density within the particle. The following equations were employed to evaluate the transient temperature change:

$$\rho C_P \frac{\partial T}{\partial t} + \nabla \cdot \boldsymbol{q} = Q \quad \text{with } Q(t) = \frac{I(t)\sigma_{abs}}{V}$$

$$\boldsymbol{q} = -k\nabla T$$

where Q represents the heat source, $I(t)$ denotes the intensity of the heating beam with a unit of W/m^2, V is the volume of a 200-nm bead, $\boldsymbol{q}$ is the conductive heat flux vector, ρ is the density of PMMA, C_P is the heat capacity of PMMA, T is the temperature, t is time, and k is the thermal conductivity. The temperature change was calculated by integrating over the bead volume. A temperature map at each time increment was generated, and we extracted the temperature profile at the center of the particle, as demonstrated in Figure S1.

The photothermal signal intensity has been found closely associated with the thermal properties of the medium, i.e., solvent. The magnitude of photothermal signals can be mathematically expressed as:[3]

$$\sum_{PT} = n\left|\frac{\partial n}{\partial T}\right|\frac{1}{C_P}$$

where n is the refractive index of the medium, $\frac{\partial n}{\partial T}$ denotes the variation of the refractive index with the temperature and is known as thermo-optic coefficient. Notably, D_2O exhibits a lower thermo-optic coefficient and a larger heat capacity compared to glycerol-d8. As a result, the thermodynamic traces shown in Figure S1b demonstrate a lower temperature rise when D_2O is used as the medium.

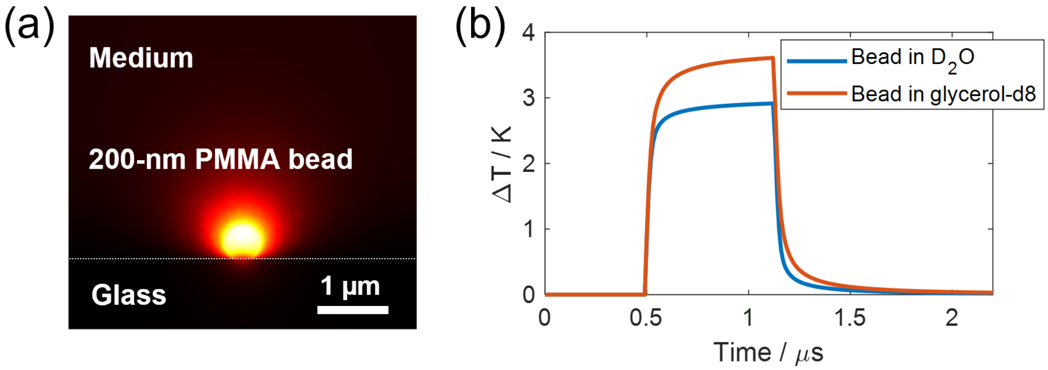

Figure S1. Thermodynamic simulation results of 200-nm PMMA beads. (a) Simulated temperature distribution of a 200-nm PMMA bead in glycerol-d8 upon heated by 1170 nm excitation beam. The shown image corresponds to the slice with the highest temperature rise, occurring at the moment when the laser pulse train is turned off. (b) Simulated temperature change throughout the process of heat accumulation and dissipation in the PMMA bead depicted in (a). Two imaging media, glycerol-d8 and D_2O were co-plotted. A smaller temperature rise of D_2O corresponds to a weaker photothermal signal intensity.

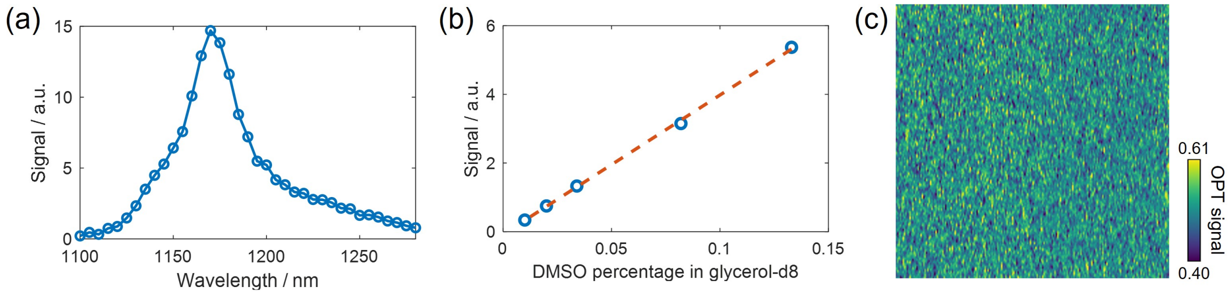

Figure S2. OPT spectroscopy of DMSO and its limit of detection in glycerol-d8. (a) OPT spectrum of pure DMSO. (b) The linear dependence of OPT signals as a function of DMSO concentrations. The five data points correspond to 1%, 2%, 3.4%, 8.2%, and 13.3% DMSO in glycerol-d8, and the OPT signals are acquired after background subtraction. The fitted linear function is expressed as $y = 40.54x - 0.07$, and the intercept -0.07 is due to experimental noises. (c) OPT image of pure glycerol-d8 at 1170 nm for noise calculation. Statistically, the limit of detection can be estimated from three times the standard deviation of background, also known as 3-Sigma. The demonstrated image contains 200 × 200 pixels, with a standard deviation of 0.042. With 3-Sigma divided by the slope of the fitted linear equation, the theoretical limit of detection is found to be 0.3% DMSO (by weight) in glycerol-d8 solutions.

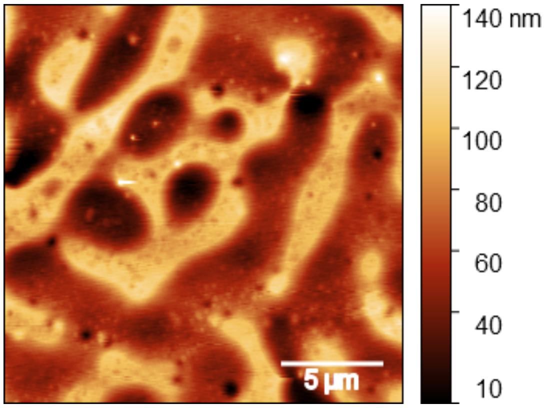

Figure S3. Representative AFM topography image of phase separation structures of PS-PMMA blend. This image captures a different area from the field-of-view presented in Fig. 3 due to challenges in colocalizing images between modalities. Nevertheless, it serves as a representative illustration of the overall morphology since the spin-coated film demonstrates uniformity across the entire sample surface. PS domains appear as irregular-shaped holes with relatively lower heights while PMMA domains are the higher matrix areas. The height difference between PS and PMMA domains is below 80 nm.

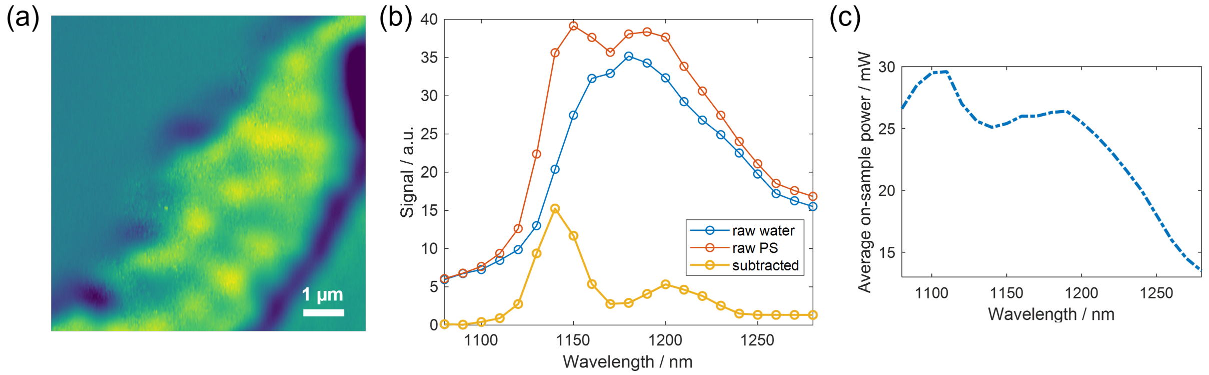

Figure S4. OPT imaging and spectroscopy of 1-µm PS bead clusters dispersed in water. (a) OPT image of 1-µm PS bead cluster at 1140 nm in a water environment. (b) OPT spectra were directly extracted from the hyperspectral image stack and normalized with laser power shown in panel (c). Raw PS signals, water signals, and a subtracted signal between PS and water were plotted together. The true PS absorption spectrum was recovered through spectroscopic analysis. (c) Average on-sample power of the SWIR excitation beam. The laser power drops when the wavelength goes beyond 1200 nm due to the drop of both laser output and transmission efficiency of the glass rods utilized for chirping purposes.

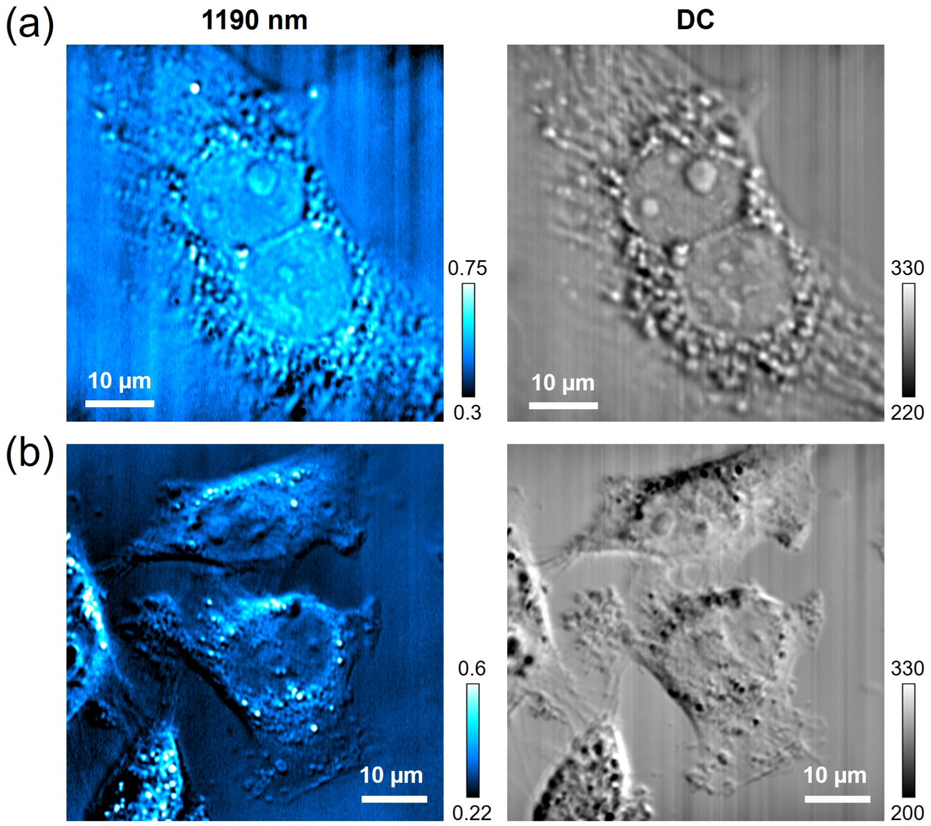

Figure S5. OPT and transmission images of OVCAR-5 cells. (a) OPT image at 1190 nm (blue) and corresponding transmission image (greyscale), with the focal plane at the cell body. (b) OPT image at 1190 nm (blue) and transmission image (greyscale), focusing on the cell bottom.

Two distinct areas of interest were investigated, representing different focal planes within the cells. The transmission images were generated using the probe beam only, with identical collection numerical apertures. Images in blue represent single-frame OPT images captured at 1190 nm, a wavelength at which both proteins and fatty acids exhibit absorption.

In Panel (a), we examine the main cell body layer, where the OPT and transmission (i.e. DC) signals do not display significant feature variation. The DC variation across the entire field of view is less than 10% and can be neglected during data processing. In Panel (b), where the focal plane is directed towards the bottom layer of cells, a substantial number of lipid droplets exhibit strong OPT signals. In contrast, these lipid droplets appear as dark spots in the transmission image due to their strong scattering properties. The specific scattering effects depend on particle size and distribution. This discrepancy between AC and DC signals highlights the potential bias introduced by scattering-based detection approaches when determining absolute chemical concentrations. Consequently, the following LASSO analysis signifies a measurement of relative concentration (semi-quantitative) within the same field of view, rather than providing absolute quantitative values.

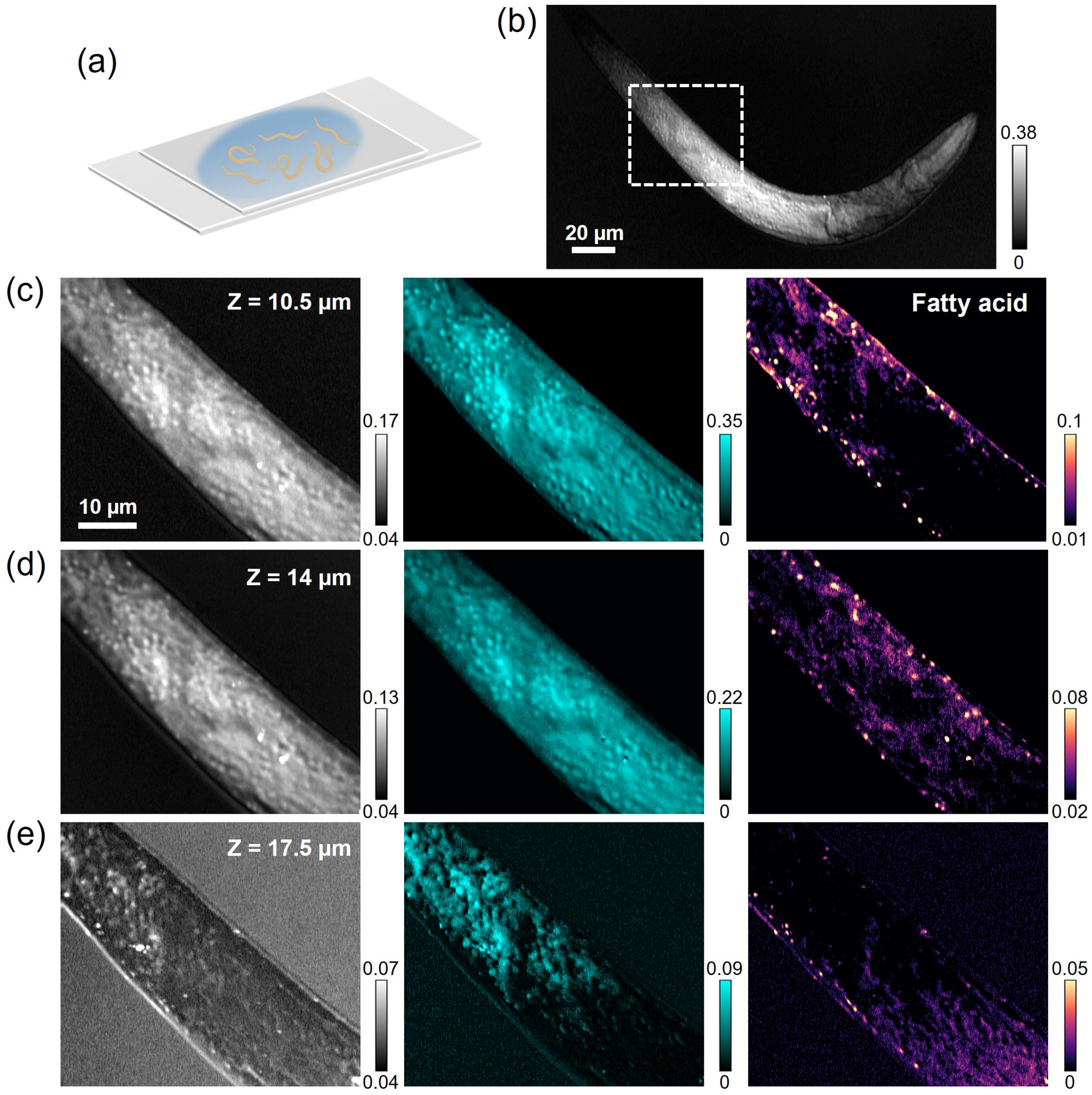

Figure S6. Depth-resolved OPT imaging and spectral unmixing on *C. elegans* worms in D_2O PBS. (a) The worms were immersed in D_2O PBS and gently sandwiched by two coverslips for imaging. (b) A full field-of-view OPT image at 1190 nm capturing an entire worm, with a zoomed-in posterior area marked for detailed analysis. (c-e) Raw OPT images of the zoomed-in section of *C. elegans* and their corresponding protein and fatty acid concentration maps at Z = 10.5 μm, Z = 14 μm, and Z = 17.5 μm, respectively. Panel (a) was created with BioRender.com released under a Creative Commons Attribution-NonCommercial-NoDerivs 4.0 International license.

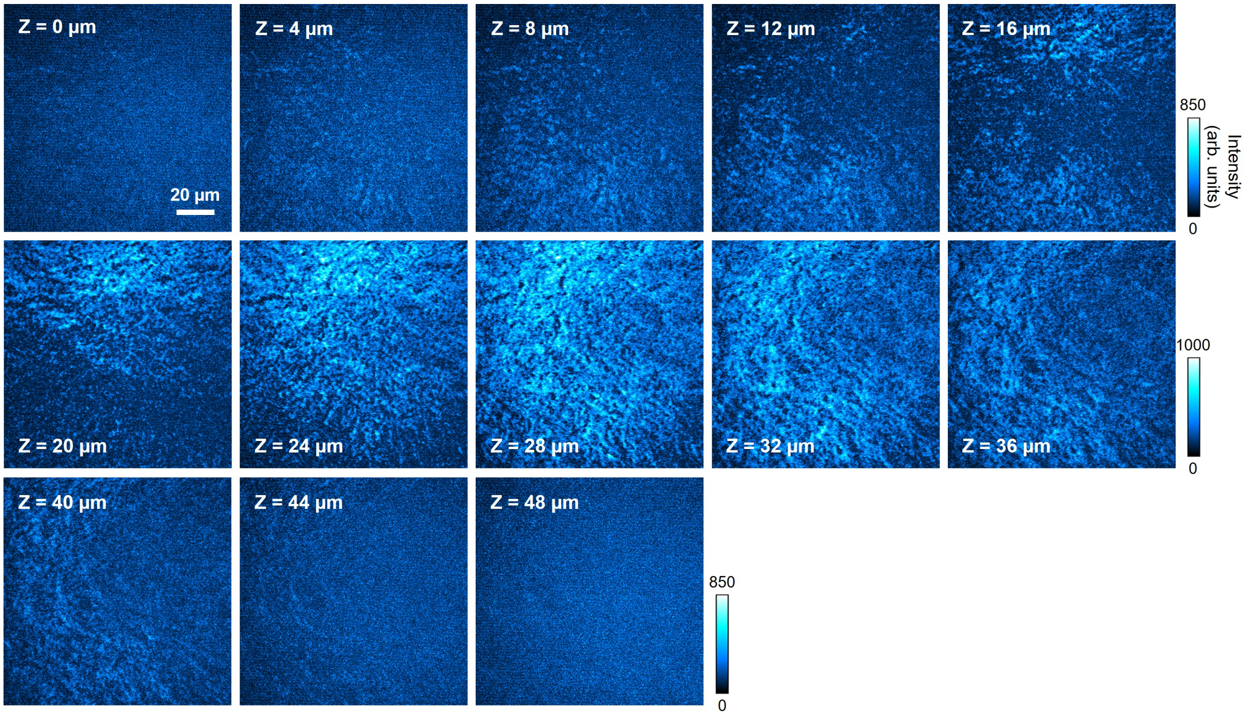

Figure S7. Mid-IR photothermal imaging of mouse brain slices to examine penetration depth. The brain slice tested has a thickness of 200 µm and was prepared together with the slice used for OPT measurements. While these slices are neighboring and expected to share similar spatial features, the exact field of view may not be identical due to challenges in precise co-localization.

During mid-IR photothermal measurements, we intentionally avoided the strongest water absorption band centered at ~1650 cm^{-1}, coinciding with the Amide I resonance. The multi-depth images were collected at 1553 cm^{-1}, corresponding to the Amide II band, which features significantly weaker water absorption. The mid-IR beam had a repetition rate of 200 kHz and a pulse duration of 500 ns. The pixel dwell time was set at 30 µs. To ensure a parallel comparison, the mid-IR experiment was conducted also in a co-propagation and forward detection configuration. The mid-IR beam and the 532 nm probe beam were colinearly focused using a reflective objective, albeit at the expense of a compromised lateral resolution due to the low numerical aperture. The results demonstrate that a penetration depth of approximately 40 µm, which is inferior to that achievable with OPT microscopy. This observation suggests that water absorption substantially reduces penetration depth and OPT microscopy outperforms mid-IR photothermal microscopy in terms of imaging deeper into biological tissues.

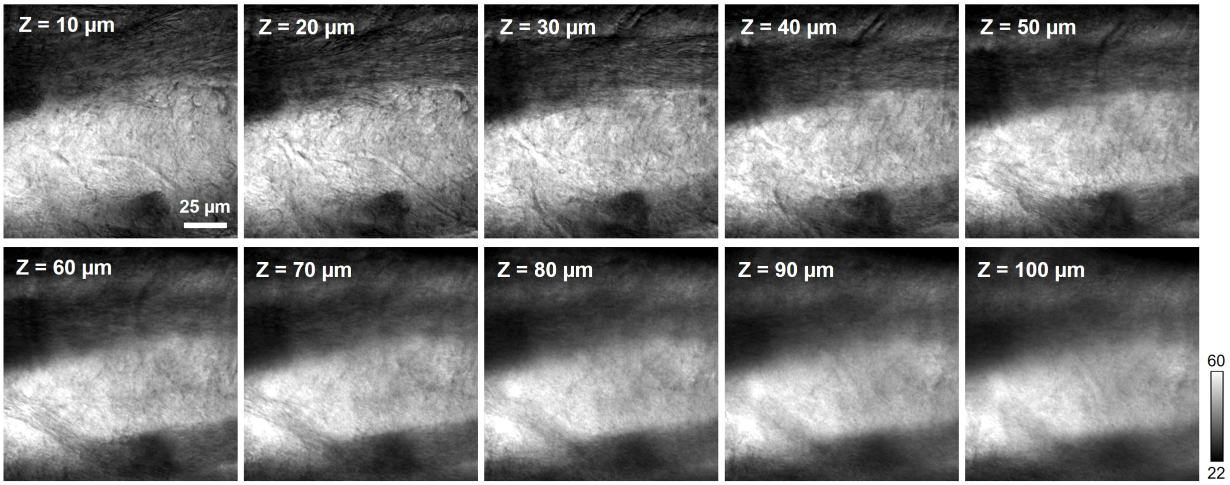

Figure S8. Transmission images of the mouse brain tissue shown in the main Fig. 7. These DC images were acquired with the 520 nm probe beam. They provide a reference for sample morphology and help evaluate potential biases in the OPT signal due to optical scattering.

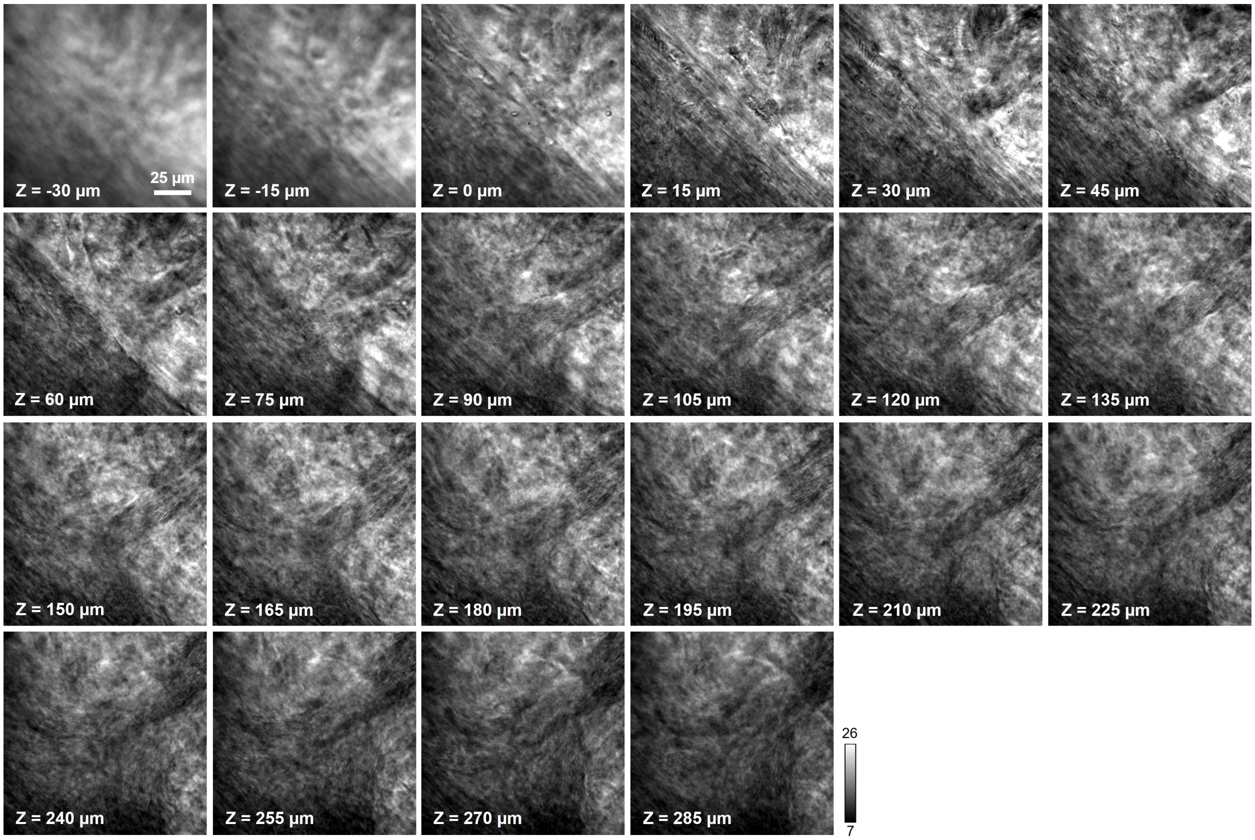

Figure S9. Depth-resolved transmission images at 1190 nm of a mouse brain slice. In contrast to the DC images in Figures S5 and S8, acquired using the 520 nm probe beam, this dataset utilized the 1190 nm pump beam, highlighting the SWIR penetration capabilities. To ensure consistency, we maintained the same on-sample average power as in OPT measurements. The $Z = -30\ \mu m$ and $Z = -15\ \mu m$ frames represent focal planes above the sample surface. The images demonstrate depths reaching up to 285 μm, constrained by the working distance (0.28 mm) of our NA = 1.2 water objective. Notably, this observation suggests that multiple scattering minimally impacts the penetration depth of the SWIR beam in comparison to the visible beam. Furthermore, this dataset enables a direct visual comparison with OPT images, showcasing OPT's superior lateral resolution and axial sectioning capabilities.

Table S1. Comparison of respective attributes of existing SWIR imaging approaches, mid-infrared photothermal (MIP) microscopy, and OPT microscopy.[4-8]

	Primary contrast	Penetration depth	Typical resolution	Limit of detection
**SWIR hyperspectral imaging**	Reflection/absorption-related optical scattering	1 – 5 mm	10 – 15 μm	Not reported
**SWIR photoacoustic microscopy (PAM)**	Ultrasonic waves from thermal expansion	~ 7 mm	~ 70 μm	~ 25 mM
**SWIR optical coherence tomography (OCT)**	Interference from optical scattering	1 – 2 mm	~ 10 μm	Not reported
**MIP**	Thermal lensing or expansion	~ 40 μm	< 500 nm	A few μM
**OPT**	Thermal lensing or expansion	~ 100 μm	~ 400 nm	A few mM

References

1 Li, Z., Aleshire, K., Kuno, M. & Hartland, G. V. Super-resolution far-field infrared imaging by photothermal heterodyne imaging. *The Journal of Physical Chemistry B* **121**, 8838-8846 (2017).

2 Velazco-Roa, M. A. & Thennadil, S. N. Estimation of optical constants from multiple-scattered light using approximations for single particle scattering characteristics. *Applied Optics* **46**, 8453-8460 (2007).

3 Gaiduk, A., Ruijgrok, P. V., Yorulmaz, M. & Orrit, M. Detection limits in photothermal microscopy. *Chemical Science* **1**, 343-350 (2010).

4 Manley, M. Near-infrared spectroscopy and hyperspectral imaging: non-destructive analysis of biological materials. *Chemical Society Reviews* **43**, 8200-8214 (2014).

5 Wang, H.-W. *et al.* Label-free bond-selective imaging by listening to vibrationally excited molecules. *Physical Review Letters* **106**, 238106 (2011).

6 Wang, L. V. & Yao, J. A practical guide to photoacoustic tomography in the life sciences. *Nature Methods* **13**, 627-638 (2016).

7 Bernstein, L. *et al.* Ultrahigh resolution spectral-domain optical coherence tomography using the 1000–1600 nm spectral band. *Biomedical Optics Express* **13**, 1939-1947 (2022).

8 Zhang, D. *et al.* Depth-resolved mid-infrared photothermal imaging of living cells and organisms with submicrometer spatial resolution. *Science Advances* **2**, e1600521 (2016).
